# Clinical Significance of Phase Angle for Assessing Quality of Life and Prognosis in Hemodialysis Patients

**DOI:** 10.3390/nu17233631

**Published:** 2025-11-21

**Authors:** Norihito Yoshida, Tatsuki Tanaka, Yusuke Suzuki, Sadamu Takahashi, Mai Hitaka, Shingo Ishii, Keisuke Yamazaki, Yasushi Ohashi

**Affiliations:** Department of Nephrology, Toho University Sakura Medical Center, Chiba 285-8741, Japan; tatsuki.tanaka@med.toho-u.ac.jp (T.T.); yusuke.suzuki@med.toho-u.ac.jp (Y.S.); sadamu.takahashi@med.toho-u.ac.jp (S.T.); mai.hitaka@med.toho-u.ac.jp (M.H.); shingo.ishii@med.toho-u.ac.jp (S.I.); keisuke.yamazaki@med.toho-u.ac.jp (K.Y.); ohashiy@med.toho-u.ac.jp (Y.O.)

**Keywords:** phase angle, bioelectrical impedance analysis, quality of life, hemodialysis, nutrition, survival, inflammation, dialysis adequacy

## Abstract

**Background/Objectives:** The phase angle (PA), derived from bioelectrical impedance analysis (BIA), reflects cellular integrity and nutritional status. Previous studies have reported associations between PA, QOL, and prognosis in hemodialysis patients; however, evidence in Japanese populations remains limited. This multicenter study aimed to confirm and extend these associations by examining the relationships of PA with QOL domains and survival outcomes in maintenance HD patients. **Methods:** In this multicenter cross-sectional study, 319 HD patients were stratified into sex-specific PA quartiles, and baseline characteristics, laboratory data, and body composition measures were compared across groups. Health-related QOL was assessed using the SF-36 and KDQOL-SF™. Associations between PA and QOL were tested with multivariable linear regression models. Survival was evaluated using Kaplan–Meier analysis with Bonferroni-adjusted pairwise comparisons. **Results:** Higher PA was significantly associated with favorable nutritional and laboratory parameters, including higher hemoglobin, albumin, creatinine, and GNRI, and lower NT-proBNP. PA also correlated positively with muscle mass and intracellular water, and inversely with the ECW/ICW ratio. Multivariable analyses showed that PA remained independently associated with several physical QOL domains, including physical functioning, role—physical, and general health, even after adjustment for dialysis adequacy (Kt/V) and inflammation (CRP). Kaplan–Meier analysis demonstrated lower survival in the lowest PA quartile, and ROC analysis identified sex-specific cutoff values for predicting mortality (4.0° for females, 4.8° for males). **Conclusions:** This multicenter confirmatory study showed that PA is independently associated with nutritional status, physical QOL, and mortality in maintenance hemodialysis patients. PA may serve as a practical, noninvasive biomarker for nutritional and functional assessment in clinical practice.

## 1. Introduction

In recent years, clinical attention has shifted beyond prognosis to include quality of life (QOL), which reflects the overall well-being and enrichment of daily living. Within nephrology, several studies have examined the relationship between chronic kidney disease (CKD) and QOL; however, few have specifically focused on patients undergoing maintenance hemodialysis (HD). In Japan, the HD population is aging, with a mean age of 70.1 years [1,2], and as malnutrition has been shown to impair QOL in older adults [3], assessing nutritional status and maintaining QOL are increasingly important in this demographic. Among various tools for evaluating malnutrition, bioelectrical impedance analysis (BIA) has attracted considerable attention as a noninvasive and rapid method to assess body composition. Phase angle (PA), a parameter derived from BIA, serves as an indicator of cellular health and nutritional status. It represents the phase shift between voltage and current as alternating current passes through body tissues, determined by the ratio of reactance (Xc) to resistance (R) [4]. Previous studies have shown that PA reflects cell membrane integrity and correlates with intracellular–extracellular water balance, body cell mass, body mass index (BMI), and survival [4], while lower PA values suggest malnutrition or underlying disease [5]. PA has been reported as a valuable biomarker for prognostic and nutritional assessment in diverse clinical populations, including patients with cancer, with chronic diseases, and on dialysis [5]. For example, Norman et al. demonstrated that low PA was strongly associated with poor prognosis in cancer patients [5,6]. Although previous studies have reported associations between PA, QOL, and survival in hemodialysis patients, evidence in Japanese populations remains scarce. Therefore, this confirmatory multicenter study in Japan was designed to validate these associations and to explore sex-specific cutoffs and non-linear survival patterns.

## 2. Materials and Methods

A multicenter, cross-sectional study was conducted from 2019 to 2022 across four chronic hemodialysis clinics: Seijinkai Mihama Hospital, Seijinkai Mihama Sakura Clinic, Seijinkai Narita Clinic, and Seijinkai Katori Clinic. During this period, 1094 patients (mean age, 68 ± 13 years; 777 males, 317 females) received hemodialysis at these facilities. Eligible participants were selected from daytime HD patients identified through electronic medical records. Inclusion criteria were adults aged 20 years or older who had been receiving dialysis treatment for 90 days or more and had maintained a stable dialysis prescription for at least 30 days at the time of recruitment. Ultimately, 368 patients who provided informed consent were included in this study. Patients were excluded if they met any of the following criteria: underwent coronary artery and/or valve surgery or experienced myocardial infarction within the past 6 months; had an unscheduled dialysis hospitalization for heart failure treatment within the past 6 months; had contraindications to bioimpedance measurement (e.g., cardiac pacemaker, joint replacement, artificial heart valve); were pregnant; or had major amputation, active malignancy, or dementia. After excluding 68 individuals with insufficient data, 319 participants were ultimately included in the analysis. For the quality of life (QOL) assessment, the Kidney Disease Quality of Life Short Form (KDQOL-SF) version 1.3, utilizing the SF-36v2^®^ Japanese version manual, was used ([Link or Citation: https://www.qualitest.jp/qol/kdqol.html] (accessed on 16 November 2025)). This study was approved by the ethics committee of Toho University Sakura Medical Center (Approval No: S24012_S21073(S18086); date: 2 August 2024) and was conducted in accordance with the Declaration of Helsinki. Informed consent was obtained from all participants. As part of clinical data collection, the following baseline data were gathered: age, sex, presence of diabetes mellitus, duration of dialysis therapy, body weight, and pre- and post-dialysis blood pressure. Additionally, blood tests and imaging examinations were routinely performed at the beginning of the month for hemodialysis management purposes, from which the following standard laboratory parameters were collected: serum albumin, blood urea nitrogen, serum creatinine, sodium, potassium, chloride, calcium, phosphorus, uric acid, glucose, C-reactive protein (CRP), hemoglobin, and intact parathyroid hormone (iPTH) levels. Dialysis adequacy was assessed using the urea reduction ratio (URR) and Kt/V urea (Kt/V), measured according to the Shinzato formula [7]. The Geriatric Nutritional Risk Index (GNRI) was calculated as (14.89 × serum albumin [g/dL]) + (41.7 × actual body weight/ideal body weight) [8]. Pre-dialysis brain natriuretic peptide (BNP) and *N*-terminal pro-B-type natriuretic peptide (NT-proBNP) levels were measured using an electrochemiluminescence immunoassay system (Cobas 8000 e801 module; Roche Diagnostics K.K., Tokyo, Japan). In this study, data were collected from routinely measured tests and the self-administered KDQOL-SF™ survey. The questionnaire was distributed during a dialysis session, with trained nurses or clinical engineers providing instructions and obtaining consent, and completed questionnaires were collected either during the same or the subsequent session. Data from blood tests, physiological function tests, and imaging examinations were extracted from electronic medical records.

### 2.1. Assessment of Body Composition and Phase Angle

Body composition and PA were measured using multi-frequency bioelectrical impedance analysis (MF-BIA) [9,10]. Measurements were performed post-dialysis utilizing a segmental MF-BIA device (InBody S10^®^; InBody Co., Ltd., Seoul, Republic of Korea), after confirmation that patients had reached their clinically determined dry weight following a 5-min rest in the supine position on a non-conductive surface. This procedure ensured that all measurements reflected the euvolemic state, minimizing the influence of extracellular fluid overload on the phase angle calculation. In the GNRI formula, the term ideal body weight refers to the theoretical weight corresponding to a BMI of 22 kg/m^2^ and is not synonymous with dry weight. The 8-electrode device determines segmental impedance across the arms, trunk, and legs at frequencies of 5, 50, 250, and 500 kHz. Phase angle at 50 kHz, commonly employed for evaluating cellular health and body composition, was calculated from resistance (R) and reactance (Xc) values obtained via the device’s software, and this 50 kHz PA value was used in all subsequent analyses. PA was calculated according to the standard formula [4]: PA (degrees) = arctangent (Xc/R) × (180/π), where Xc represents reactance (Ω) and R represents resistance (Ω).

### 2.2. Statistical Analysis

We performed all statistical analyses using JMP Pro version 17 (SAS Institute Inc.). Continuous variables are summarized as median [interquartile range (IQR, 25th–75th percentile)], and categorical variables as number (%). Normality of phase angle (PA) distribution was assessed using the Shapiro–Wilk (W = 0.987, *p* = 0.008) and Anderson–Darling (A^2^ = 1.113, *p* = 0.009) tests; both indicated non-normal distribution. To address potential sex differences in PA levels and ensure balanced group sizes for non-normally distributed data, participants were stratified into quartiles based on sex-specific PA cutoffs. These cutoffs were determined by ranking PA values separately for males and females (Q1 [lowest]: males ≤ 4.5°, females ≤ 4.0°; Q2: males 4.6–5.3°, females 4.1–4.8°; Q3: males 5.4–6.2°, females 4.9–5.2°; Q4 [highest]: males ≥ 6.3°, females ≥ 5.3°). This stratification yielded group sizes of *n* = 70 (23.7%), *n* = 75 (25.4%), *n* = 78 (26.4%), and *n* = 72 (24.4%) for Q1–Q4, respectively. These quartiles were used in subsequent analyses. Baseline characteristics, laboratory findings, medication usage, GNRI, and body composition measures were compared across PA quartiles using the Kruskal–Wallis test for continuous variables and the Pearson chi-square test for categorical variables. To examine the association between continuous PA values and quality of life (QOL) scores derived from the SF-36 and KDQOL-SF, we developed three sequential linear regression models. Model 1 was a univariable model with PA as the sole predictor. Model 2 adjusted for age, sex, and pre-dialysis *N*-terminal pro-B-type natriuretic peptide (NT-proBNP). Model 3 incorporated the covariates from Model 2 plus HD duration, diabetes mellitus (DM), history of cardiovascular disease (CVD), smoking status, GNRI, single-pool Kt/V urea (Kt/V), and pre-dialysis creatinine (Cr), hemoglobin (Hb), and C-reactive protein (CRP). The association between PA quartiles and all-cause mortality was initially assessed using the Kaplan–Meier method, with group differences compared via the log-rank test, using Q1 as the reference. Subsequently, multivariable analyses employed parametric survival models assuming a Weibull distribution to estimate hazard ratios (HRs) and 95% confidence intervals (CIs). The Weibull model was selected over the standard Cox proportional hazards model because it provided better fit indices (lower AIC/BIC) and allowed direct estimation of baseline hazard functions, which is advantageous when the number of events is limited. Receiver operating characteristic (ROC) analyses were also performed to determine sex-specific phase angle cutoff values for mortality prediction. The 95% confidence intervals (CIs) for the areas under the ROC curves (AUCs) were calculated using the DeLong method. We fitted three sequential models: Model 1 included only PA quartiles; Model 2 adjusted for age, sex, and NT-proBNP; and Model 3 further adjusted for HD duration, DM, CVD, smoking status, Cr, Hb, GNRI, Kt/V, and CRP. Across all analyses, a two-sided *p* < 0.05 was considered statistically significant. Continuous variables are presented as median [IQR, 25th–75th percentile], and categorical variables as number (%); these conventions apply to all tables and figures unless otherwise specified.

### 2.3. Survival Analysis

Survival was defined as the time from baseline assessment to death from any cause or censoring at the end of follow-up. Follow-up time was calculated from baseline assessment to death or administrative censoring on 31 March 2023, and the median follow-up was 41.2 months (IQR: 32.6–45.3), yielding 1185.5 person-years of observation. During follow-up, 38 deaths occurred. Pairwise between-quartile comparisons were additionally performed using log-rank tests. To control multiple testing across six pairwise comparisons (*m* = 6), statistical significance was defined as *p* < 0.0083 (Bonferroni correction), and adjusted *p*-values were reported as min (1, *p* × *m*).

## 3. Results

### 3.1. Baseline Characteristics and Clinical Parameters

Patients’ baseline characteristics and clinical parameters were compared across PA quartiles (Table 1). Age was significantly higher in the lowest PA quartile (Q1) and progressively lower in higher PA quartiles, indicating a strong inverse relationship between PA and age (median [IQR]: Q1, 73.5 [69.0–79.0] years; Q4, 55.0 [48.0–63.0] years; *p* < 0.001). HD duration showed a decreasing trend across quartiles, although this did not reach statistical significance (*p* = 0.051), while the proportion of males, history of CVD, DM, and smoking history did not differ significantly among quartiles. However, BMI increased significantly with higher PA (Q1: 20.06 [18.42–22.22]; Q4: 24.50 [21.90–27.37]; *p* < 0.0001), as did GNRI (Q1: 90.3 [84.8–97.0]; Q4: 101.7 [96.0–107.0]; *p* < 0.001). Several laboratory markers were also significantly associated with PA, including hemoglobin, albumin, blood urea nitrogen, creatinine, uric acid, potassium, chloride, HDL cholesterol, NT-proBNP, and Kt/V. Notably, NT-proBNP levels were substantially lower in higher PA quartiles (Q1: 8825 [4928–19,625] pg/mL; Q4: 2000 [1133–3760] pg/mL; *p* < 0.001), suggesting that better nutritional and fluid status may correspond to reduced cardiac stress.

### 3.2. Body Composition Parameters by PA Quartiles

Body composition indices are summarized by PA quartile in Table 2. Total body water (TBW), extracellular water (ECW), and intracellular water (ICW) all increased progressively with higher PA quartiles, with the most pronounced increase observed in ICW (17.8 L in Q1 to 22.7 L in Q4, *p* < 0.001), indicating better preservation of cellular water content in patients with higher PA. Muscle mass and fat-free mass were also significantly greater in higher PA quartiles, while fat mass increased moderately across groups. In contrast, the extracellular-to-intracellular water (ECW/ICW) ratio declined steadily with higher PA (Q1: 0.704; Q4: 0.633, *p* < 0.001), reflecting improved cellular integrity and fluid balance. Collectively, these findings demonstrate that higher PA is associated with a more favorable body composition profile, characterized by greater muscle mass, higher ICW, and lower relative extracellular expansion.

### 3.3. PA and Health-Related QOL Relationship with Multivariate Analysis

Table 3 and Table 4 summarize QOL domain scores derived from the SF-36 and KDQOL-SF questionnaires. Across quartiles, higher PA was consistently linked to better physical domains, particularly physical functioning, role—physical, and bodily pain, as well as vitality and role—emotional. In the KDQOL-SF, higher PA was associated with more favorable scores in the effects of kidney disease and work status domains. Conversely, no significant associations were observed in mental health, sleep, or social support. These findings indicate that PA primarily reflects physical rather than psychosocial QOL aspects in HD patients.

**Figure 1:**
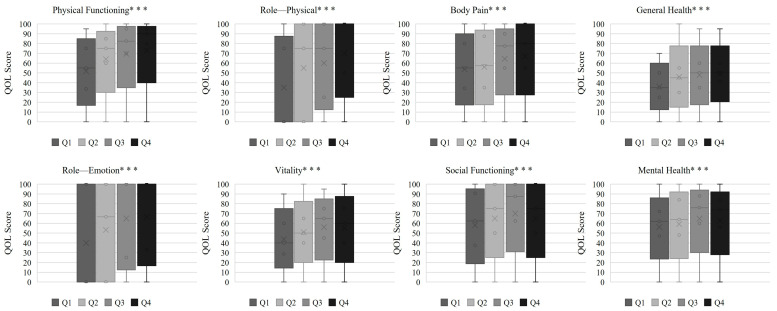
SF-36 subscale quality of life scores across phase angle quartiles. Note: Scores are presented as medians. PA quartiles were defined using sex-specific cutoffs. *** *p* < 0.001.

**Figure 2:**
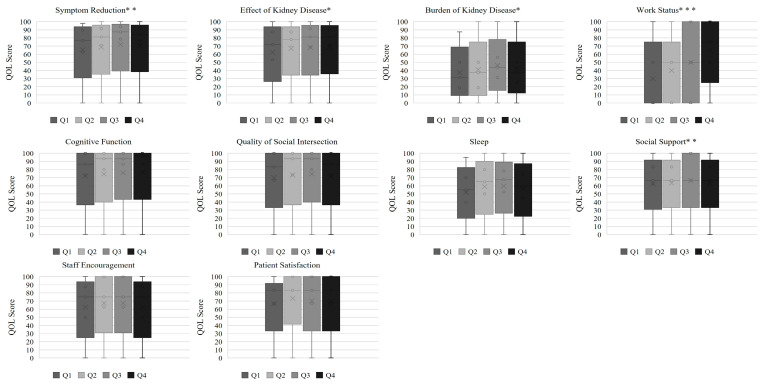
KDQOL-SF subscale quality of life scores across phase angle quartiles. Note: Scores are presented as medians. PA quartiles were defined using sex-specific cutoffs. * *p* < 0.05, ** *p* < 0.01, *** *p* < 0.001.

Figure 1 and Figure 2 show the distributions of SF-36 and KDQOL-SF™ subscale scores, respectively, across sex-specific PA quartiles.

In the SF-36 domains (Figure 1), patients with higher PA exhibited progressively better scores for physical functioning, role—physical, bodily pain, general health, vitality, and role—emotional. Social functioning and mental health also improved with increasing PA, although to a lesser extent. In the KDQOL-SF domains (Figure 2), higher PA was associated with better scores for the effects of kidney disease and work status, whereas sleep, cognitive function, and patient satisfaction showed no significant differences. These patterns indicate that PA is more strongly related to physical aspects of QOL than to psychosocial or kidney-disease-specific domains.

**Figure 3:**
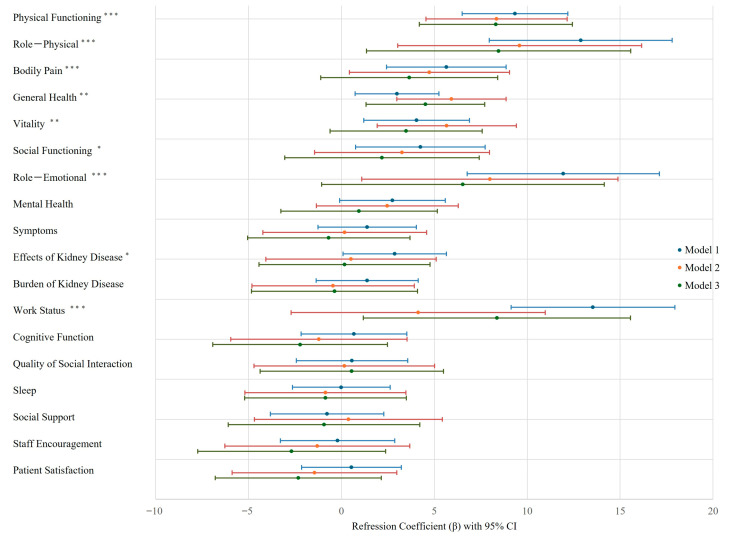
Forest plot depicting the associations between phase angle and QOL. Note: Associations between phase angle (PA) and 18 QOL domains are shown. Regression coefficients (β) with 95% confidence intervals (CIs) are presented for three models, described in the Methods section. Asterisks denote significance: * *p* < 0.05, ** *p* < 0.01, *** *p* < 0.001.

To evaluate the association between PA and QOL scores, multivariable linear regression analyses were conducted (Supplementary Table S1). In the unadjusted model (Model 1), PA showed significant positive associations with several SF-36 subscales, including physical functioning, role—physical, bodily pain, general health, vitality, social functioning, and role—emotional. In addition, PA was positively associated with the KDQOL-SF subscales for the effects of kidney disease and work status (*p* < 0.05 for all). After adjustment for age, sex, and baseline NT-proBNP (Model 2), PA remained significantly associated with SF-36 domains of physical functioning (β = 8.35, *p* < 0.001), role—physical (β = 9.59, *p* = 0.005), bodily pain (β = 4.74, *p* = 0.032), general health (β = 5.92, *p* < 0.001), vitality (β = 5.67, *p* = 0.003), and role—emotional (β = 7.99, *p* = 0.024). In the fully adjusted model (Model 3), which included dialysis duration, diabetes mellitus, cardiovascular disease, smoking status, serum creatinine, hemoglobin, GNRI, Kt/V, and CRP, PA remained independently associated with SF-36 physical functioning (β = 8.31, *p* < 0.001), role—physical (β = 8.46, *p* = 0.020), general health (β = 4.52, *p* = 0.006), and the KDQOL-SF work status domain (β = 8.37, *p* = 0.023). No significant associations were observed for other QOL domains (Figure 3).

### 3.4. Kaplan–Meier Survival Analysis

**Figure 4:**
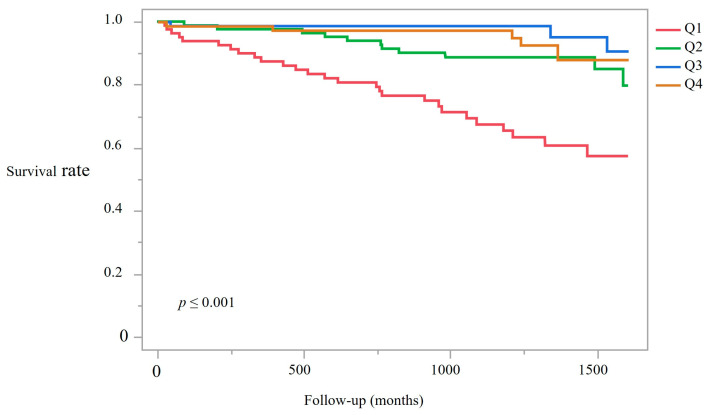
Kaplan–Meier survival curves stratified by PA quartiles. Note: Survival differed significantly among quartiles (log-rank test, *p* < 0.001). Follow-up was censored on 31 March 2023; 38 deaths occurred (median follow-up: 41.2 months). Log-rank test, *p* < 0.001.

During a median follow-up of 41.2 months (IQR: 32.6–45.3), a total of 38 deaths occurred among 319 patients, corresponding to 1185.5 person-years of observation. The numbers of deaths were 17, 11, 6, and 4 in Q1–Q4, respectively. Kaplan–Meier survival analysis revealed significant differences among quartiles (log-rank test: χ^2^ = 38.63, *p* < 0.001; Figure 4), and pairwise log-rank tests with Bonferroni correction (α = 0.05/6 = 0.0083) were additionally performed to assess survival differences between quartiles. After correction, only the comparison between Q1 and Q4 remained statistically significant, indicating that the survival benefit was primarily driven by the highest PA group.

Patients in the lowest PA quartile (Q1) had substantially poorer survival compared with those in higher quartiles. Mortality rates per 100 person-years were highest in Q1 (15.6) and lowest in Q3 (2.1). Although the rate in Q4 was slightly higher at 3.2, this was still substantially lower than Q1 (Supplementary Table S2).

ROC curve analysis identified optimal sex-specific PA cutoff values for predicting mortality risk. In females, the area under the curve (AUC) was 0.932 (95% CI, 0.87–0.99)**,** indicating excellent discriminative ability, with an optimal cutoff of 4.0° (sensitivity 100%, specificity 82.3%; Supplementary Figure S1). In males, the AUC was 0.722 (95% CI, 0.60–0.84), with an optimal cutoff of 4.8° (sensitivity 69.2%, specificity 74.8%; Supplementary Figure S2).

Parametric survival analysis assuming a Weibull distribution confirmed the prognostic significance of PA quartiles. Compared with Q1, the fully adjusted hazard ratios (Model 3; adjusted for age, sex, NT-proBNP, dialysis duration, diabetes mellitus, cardiovascular disease, smoking status, serum creatinine, hemoglobin, GNRI, Kt/V, and CRP) were significantly lower in higher quartiles: Q2, HR 3.59 (95% CI 1.54–8.35, *p* = 0.002); Q3, HR 11.70 (95% CI 4.10–33.37, *p* < 0.001); and Q4, HR 3.96 (95% CI 1.74–8.99, *p* = 0.001) (Supplementary Table S3).

Collectively, these results demonstrate the independent predictive value of PA for mortality risk in chronic HD patients and underscore the potential clinical relevance of sex-specific PA thresholds (4.0° for females and 4.8° for males) for identifying high-risk individuals.

## 4. Discussion

This study comprehensively evaluated the associations of PA with health-related QOL and prognosis in patients undergoing maintenance hemodialysis. PA, measured by BIA, serves as a surrogate marker of cellular integrity and body composition [11]. Previous studies have reported associations of PA with muscle mass, nutritional status, QOL, and survival [12]. Although similar relationships have been demonstrated previously, the present study provides confirmatory evidence from a multicenter Japanese cohort.

In particular, the application of sex-specific quartiles and ROC-derived cutoff values offers practical stratification insights for clinical use. The key finding of this study is that PA was consistently associated with three major clinical domains in hemodialysis patients: (1) nutritional and body composition indicators; (2) health-related QOL, particularly its physical components; (3) all-cause mortality. These results suggest that PA serves as a central integrative biomarker linking nutritional status, functional capacity, and prognosis. Although protein-energy wasting (PEW) and reduced physical activity are common in dialysis patients and may partly explain these relationships, our findings indicate that PA itself serves as a practical and comprehensive marker connecting malnutrition, impaired QOL, and higher mortality risk. At baseline, PA was positively associated with hemoglobin, albumin, creatinine, and GNRI, while lower PA was accompanied by lower HDL-C and higher uric acid levels. These findings indicate that higher PAs reflect better hematologic and nutritional status, consistent with preserved protein-energy reserves and greater muscle mass. Conversely, lower PAs may capture features of malnutrition, anemia, and metabolic disturbances, consistent with prior studies linking poor nutritional indices to adverse outcomes in dialysis populations [12,13,14]. Beyond laboratory parameters, PA showed a strong inverse correlation with the extracellular water to intracellular water ECW/ICW ratio, supporting the interpretation that reduced PA reflects relative expansion of extracellular fluid due to diminished cell mass and impaired membrane integrity.

This study adds evidence beyond previous reports in several key respects. Compared with previous reports [12], the present multicenter study provides additional insights in several respects. First, it included participants from multiple dialysis centers in Japan, enhancing the generalizability of the findings. Second, it comprehensively evaluated 18 domains of QOL using both the SF-36 and KDQOL instruments, thereby capturing physical, mental, and social dimensions of health. Third, sex-specific quartile and ROC analyses were conducted to propose clinically relevant cutoff values for patient stratification. These aspects extend the existing evidence regarding the clinical utility of PA in hemodialysis patients. Thus, PA is not only a nutritional marker but also an indicator of body composition quality and fluid balance, both of which are highly relevant in the dialysis population. Beyond its established role as a marker of cellular integrity and nutritional status, PA may also reflect physical function and frailty status in HD patients. Recent studies have shown that lower PA is associated with decreased ability in activities of daily living (ADL) and with pre-frailty conditions. Li et al. [8] demonstrated a significant association between low PA and disability in ADL, while Yang et al. [15] reported that combining PA with objective physical function assessments could predict pre-frailty more effectively. These findings suggest that PA captures not only cellular-level health but also functional decline, thereby providing an integrative biomarker that bridges nutritional, physiological, and functional domains in maintenance hemodialysis patients. A key finding of this study is that PA was independently associated with physical QOL domains—including physical functioning, role—physical, and general health—even after multivariable adjustment. In contrast, PA showed weaker associations with mental, social, and kidney-disease-specific domains, suggesting that PA primarily reflects physical rather than psychosocial health [16]. Protein-energy wasting (PEW), common in dialysis patients, likely mediates this relationship [17], and uremia-related anorexia, altered taste, depression, aging, and reduced oral intake all contribute to PEW [8,18]. Moreover, reduced physical activity during dialysis, prolonged sedentary time, and muscle catabolism accelerate sarcopenia and frailty [18,19], leading to reduced mobility, falls, fractures, and ultimately impaired physical QOL [20,21]. Importantly, the associations between PA and physical QOL remained significant even after adjustment for dialysis adequacy (Kt/V) and inflammation (CRP). While Kt/V reflects dialysis efficiency, its interpretation is complex because higher values often occur in leaner patients. In contrast, CRP is a well-established predictor of reduced QOL, as elevated levels promote muscle catabolism, appetite loss, and fatigue, as demonstrated in the PIVOTAL trial [22]. The persistence of these associations suggests that PA captures additional aspects of physiological reserve beyond dialysis adequacy and inflammation.

Chronic inflammation reflected by elevated CRP may promote muscle protein breakdown, reduce appetite, and increase fatigue, directly impairing physical functioning and overall health. The persistence of significant associations between PA and physical QOL even after adjustment for Kt/V and CRP suggests that PA captures additional dimensions of physiological reserve beyond dialysis adequacy and inflammation. A key aspect of this study is the identification of sex-specific cutoff values and the observation of a non-linear association between PA and survival. ROC analysis demonstrated excellent predictive performance in females (AUC 0.932, cutoff 4.0°) and moderate performance in males (AUC 0.722, cutoff 4.8°), likely reflecting sex differences in muscle mass and body composition. Survival analysis indicated that PA was associated with improved prognosis, although the apparent advantage of the third quartile should be interpreted with caution due to the limited number of events. Rather than a gradual risk gradient, a marked decline in PA appears to be associated with a disproportionate increase in mortality risk, indicating that PA may act as a threshold-based biomarker reflecting critical cellular and nutritional deterioration. Taken together with our pairwise, Bonferroni-adjusted log-rank results and the parametric survival modeling, these findings support a threshold-like (non-linear) association in which marked reductions in PA—rather than incremental changes across the entire range—are disproportionately associated with higher mortality risk.

Overall, PA represents more than a nutritional marker; it is a clinically useful, noninvasive, and reproducible biomarker that reflects body composition, cellular integrity, and prognosis in patients receiving maintenance hemodialysis [23,24,25]. However, its prognostic significance may vary across ethnicities and dialysis practices, emphasizing the need for validation in larger and more diverse populations. Moreover, PA is potentially modifiable through nutritional and exercise interventions, both of which have been shown to improve PA and may, in turn, enhance QOL and survival [26,27,28,29]. Future studies should aim to establish sex-specific cutoff values, clarify non-linear prognostic associations, and determine whether interventions that improve PA translate into better outcomes for hemodialysis patients.

Given the limited number of events, the sex-specific ROC performance observed in this study may be overly optimistic; therefore, both internal validation (e.g., bootstrap correction, DeLong 95% CIs) and external validation in independent cohorts are warranted before clinical application. Additionally, future research integrating PA with objective measures of muscle strength and physical activity may further elucidate the physiological pathways linking nutrition, function, and QOL in this population.

### Limitations

This study has several limitations. First, its cross-sectional design precludes establishing a causal relationship between PA and QOL. Although we employed a parametric Weibull survival model to estimate hazard ratios—chosen for its better model fit (lower AIC/BIC) and stability with a limited number of events—the relatively short follow-up period and small number of deaths still preclude robust longitudinal inference. Therefore, our survival findings should be interpreted as exploratory rather than confirmatory. The sex-specific ROC analyses were based on a small number of events and may be susceptible to overfitting; accordingly, these cutoffs should be considered exploratory pending external validation. Second, PA measurements were performed at a single time point, which does not account for potential variability over time due to changes in fluid status or nutritional condition. Furthermore, functional performance parameters such as handgrip strength or physical activity were not assessed in this study. Therefore, the observed association between PA and QOL should be interpreted as correlational rather than causal. Third, although PA was associated with body composition and nutrition-related parameters, residual confounding by unmeasured factors such as physical activity levels or psychosocial stress may remain despite multivariable adjustment. Fourth, the study relied on self-reported questionnaires for QOL assessment, which may have introduced response bias. Fifth, the number of events in the higher PA quartiles (particularly Q3 and Q4) was relatively small, which may have influenced the interpretation of survival differences, and consequently the apparent advantage of Q3 over other quartiles should be interpreted with caution due to potential statistical instability. Sixth, the optimal PA cutoff values derived from ROC analyses may not be generalized to other populations and should be validated in external cohorts. Finally, as this was a confirmatory study conducted exclusively among Japanese HD patients, potential selection bias should be acknowledged, and the generalizability of the findings to other ethnic or healthcare settings may be limited. Additionally, as this study was conducted at four affiliated dialysis centers, potential selection bias and center-specific practice patterns cannot be excluded. Residual confounding due to unmeasured factors such as physical activity, dietary intake, or psychosocial variables may also remain despite multivariable adjustment.

## 5. Conclusions

This multicenter confirmatory study showed that PA is independently associated with the physical domains of QOL and predicts all-cause mortality in maintenance hemodialysis patients. These associations remained significant even after adjustment for dialysis adequacy (Kt/V) and inflammation (CRP), suggesting that PA provides information complementary to conventional clinical parameters. By confirming these associations in a Japanese cohort, this study reinforces the potential utility of PA as a practical, complementary, and integrative biomarker for nutritional and functional assessment in hemodialysis care.

## Figures and Tables

**Table 1:** Baseline characteristics of study participants stratified by phase angle quartiles.

	**Q1 (*n* = 70)**	**Q2 (*n* = 75)**	**Q3 (*n* = 78)**	**Q4 (*n* = 72)**	***p*-Value**
PA in males PA in females	≤4.5° ≤4.0°	4.6–5.3° 4.1–4.8°	5.4–6.2° 4.9–5.2°	≥6.3° ≥5.3°	
Age (years)	73.5 (69.0–79.0)	70.0 (59.0–76.0)	66.5 (54.8–72.3)	55.0 (48.0–63.0)	<0.001
Male (%)	34 (48.6%)	41 (54.7%)	40 (51.3%)	43 (59.7%)	0.68
HD duration (months)	82.5 (42.8–221.3)	68.0 (37.0–158.0)	68.5 (35.8–112.5)	60.0 (26.3–118.5)	0.05
History of CVD (%)	28 (40.0%)	23 (30.7%)	35 (44.9%)	23 (31.9%)	0.32
History of DM (%)	31 (44.3%)	36 (48.0%)	37 (47.4%)	27 (37.5%)	0.55
Smoking (%)	18 (25.7%)	21 (28.0%)	29 (37.2%)	16 (22.2%)	0.21
BMI (kg/m^2^)	20.1 (18.4–22.2)	21.6 (19.9–23.6)	22.7 (20.6–25.5)	24.5 (21.9–27.4)	<0.001
% of fluid removal	4.9 (4.0–6.0)	5.1 (4.2–5.7)	5.3 (4.0–6.0)	5.4 (4.4–6.2)	0.32
Hb (g/dL)	11.1 (10.5–11.5)	11.2 (10.6–11.7)	11.4 (10.8–12.0)	11.7 (10.9–12.3)	<0.001
Alb (g/dL)	3.5 (3.3–3.7)	3.6 (3.3–3.7)	3.6 (3.5–3.8)	3.7 (3.5–3.9)	<0.001
BUN (mg/dL)	53.9 (46.5–60.8)	57.0 (46.0–72.9)	56.1 (46.8–65.9)	65.9 (55.7–73.9)	<0.001
Cr (mg/dL)	8.70 (7.51–9.89)	9.57 (8.62–10.92)	10.75 (9.42–11.92)	12.69 (10.54–13.73)	<0.001
Uric acid (mg/dL)	6.9 (6.4–7.8)	7.3 (6.6–8.2)	7.8 (7.2–8.4)	8.5 (7.6–9.4)	<0.001
Na (mEq/L)	139 (138–140)	139 (138–141)	139 (138–140)	139 (137–141)	0.86
K (mEq/L)	4.6 (4.2–5.1)	4.8 (4.5–5.3)	4.8 (4.4–5.3)	4.9 (4.4–5.4)	0.045
Cl (mEq/L)	104 (102–106)	104 (102–106)	103 (102–105)	103 (100–104)	0.003
HDL-C (mg/dL)	50.0 (43.0–61.3)	55.0 (43.0–65.0)	46.0 (38.0–61.0)	45.0 (37.0–55.8)	0.011
iPTH (pg/mL)	149 (94–219)	172 (100–221)	151 (113–230)	170 (107–229)	0.56
β2MG (mg/L)	26.0 (23.0–29.5)	25.5 (22.4–29.7)	26.3 (23.8–28.5)	27.0 (24.0–30.5)	0.40
CRP (mg/dL)	0.15 (0.07–0.36)	0.10 (0.04–0.28)	0.07 (0.04–0.23)	0.14 (0.06–0.31)	0.06
NT-proBNP (pg/mL)	8825 (4928–19,625)	3690 (1850–7650)	2930 (1813–5763)	2000 (1133–3760)	<0.001
Kt/V urea	1.80 (1.69–2.07)	1.92 (1.72–2.17)	1.87 (1.64–2.06)	1.72 (1.59–1.93)	0.003
GNRI	90.3 (84.8–97.0)	92.0 (87.7–98.1)	96.9 (92.0–104.0)	101.7 (96.0–107.0)	<0.001

Note: Categorical variables are presented as number (%). Patients were stratified into sex-specific PA quartiles.

**Table 2:** Body composition parameters by PA quartile.

	Q1 (*n* = 70)	Q2 (*n* = 75)	Q3 (*n* = 78)	Q4 (*n* = 72)	*p*-Value
PA in males PA in females	≤4.5° ≤4.0°	4.6–5.3° 4.1–4.8°	5.4–6.2° 4.9–5.2°	≥6.3° ≥5.3°	
TBW (L)	29.9 (24.3–33.7)	31.5 (26.4–35.4)	34.9 (28.3–40.5)	36.2 (29.7–41.0)	<0.001
ECW (L)	12.2 (9.9–13.7)	12.4 (10.5–13.7)	13.5 (11.1–15.5)	13.5 (11.5–15.3)	0.002
ICW (L)	17.8 (14.4–20.1)	19.1 (15.9–21.4)	21.5 (17.4–25.0)	22.7 (18.4–26.1)	<0.001
Muscle mass (kg)	8.4 (6.7–9.0)	8.9 (7.4–9.9)	10.0 (7.8–11.3)	10.4 (8.5–11.9)	<0.001
Fat-free mass (kg)	44.0 (35.6–48.3)	46.6 (39.5–51.3)	51.5 (40.9–58.4)	53.7 (43.9–61.0)	<0.001
Fat mass (kg)	12.2 (8.1–16.9)	12.5 (9.3–20.3)	14.6 (10.9–21.2)	17.8 (12.8–24.2)	< 0.001
ECW/ICW ratio	0.704 (0.694–0.721)	0.677 (0.667–0.693)	0.659 (0.646–0.672)	0.633 (0.619–0.651)	<0.001

Note: Values are presented as medians. PA quartiles were defined using sex-specific cutoffs.

**Table 3:** SF-36 quality of life scores according to phase angle quartiles.

	Q1 (*n* = 70)	Q2 (*n* = 75)	Q3 (*n* = 78)	Q4 (*n* = 72)	*p*-Value
PA in Males PA in Females	≤4.5° ≤4.0°	4.6–5.3° 4.1–4.8°	5.4–6.2° 4.9–5.2°	≥6.3° ≥5.3°	
Physical Functioning	55.0 (33.8–75.0)	75.0 (60.0–85.0)	82.5 (70.0–95.0)	90.0 (80.0–95.0)	<0.001
Role—Physical	0.0 (0.0–75.0)	75.0 (0.0–100.0)	75.0 (25.0–100.0)	100.0 (50.0–100.0)	<0.001
Bodily Pain	55.0 (34.4–80.0)	57.5 (35.0–87.5)	77.5 (55.0–90.0)	80.0 (55.0–100.0)	<0.001
General Health	35.0 (25.0–50.0)	45.0 (30.0–55.0)	50.0 (35.0–60.0)	50.0 (41.3–60.0)	<0.001
Vitality	40.0 (28.8–60.0)	50.0 (40.0–65.0)	65.0 (45.0–75.0)	60.0 (40.0–75.0)	<0.001
Social Functioning	62.5 (37.5–90.6)	75.0 (50.0–100.0)	87.5 (62.5–100.0)	75.0 (50.0–100.0)	0.002
Role—Emotional	0.0 (0.0–100.0)	66.7 (0.0–100.0)	100.0 (25.0–100.0)	100.0 (33.3–100.0)	<0.001
Mental Health	62.0 (47.0–72.0)	64.0 (48.0–84.0)	76.0 (60.0–88.0)	74.0 (56.0–84.0)	0.002

Note: Scores are presented as medians. PA quartiles were defined using sex-specific cutoffs.

**Table 4:** Kidney-disease-specific QOL scores (KDQOL-SF) according to phase angle quartiles.

	Q1 (*n* = 70)	Q2 (*n* = 75)	Q3 (*n* = 78)	Q4 (*n* = 72)	*p*-Value
PA in Males PA in Females	≤4.5° ≤4.0°	4.6–5.3° 4.1–4.8°	5.4–6.2° 4.9–5.2°	≥6.3° ≥5.3°	
Symptom Reduction	77.1 (62.5–89.6)	81.3 (70.8–91.7)	87.5 (78.6–93.8)	84.4 (77.1–91.7)	0.007
Effects of Kidney Disease	71.9 (53.1–87.5)	78.1 (68.8–87.5)	81.3 (68.8–91.4)	81.3 (71.9–90.6)	0.015
Burden of Kidney Disease	31.3 (18.8–50.0)	37.5 (18.8–50.0)	43.8 (31.3–56.3)	37.5 (25.0–50.0)	0.030
Work Status	0.0 (0.0–50.0)	50.0 (0.0–50.0)	50.0 (0.0–100.0)	75.0 (50.0–100.0)	<0.001
Cognitive Function	86.7 (73.3–100.0)	93.3 (80.0–100.0)	93.3 (86.7–100.0)	96.7 (86.7–100.0)	0.180
Quality of Social Interaction	83.3 (66.7–100.0)	93.3 (73.3–100.0)	93.3 (80.0–100.0)	86.7 (73.3–100.0)	0.060
Sleep	55.0 (40.0–70.0)	65.0 (50.0–80.0)	67.5 (52.5–78.1)	60.0 (45.0–74.4)	0.060
Social Support	66.7 (62.5–83.3)	66.7 (66.7–83.3)	66.7 (66.7–100.0)	66.7 (66.7–83.3)	0.046
Staff Encouragement	75.0 (50.0–100.0)	75.0 (62.5–100.0)	75.0 (62.5–100.0)	75.0 (50.0–100.0)	0.070
Patient Satisfaction	83.3 (66.7–100.0)	83.3 (83.3–100.0)	83.3 (66.7–100.0)	83.3 (66.7–100.0)	0.190

Note: Scores are presented as medians. PA quartiles were defined using sex-specific cutoffs.

## Data Availability

The datasets generated and analyzed during the current study are available from the corresponding author on reasonable request.

## Supplementary Materials

The following supporting information can be downloaded at https://www.mdpi.com/article/10.3390/nu17233631/s1: Figure S1: Receiver operating characteristic curve for sex-specific phase angle cutoff values predicting mortality in females; Figure S2: Receiver operating characteristic curve for sex-specific phase angle cutoff values predicting mortality in males; Table S1: Multivariable linear regression analysis of the association between phase angle and QOL scores; Table S2: Mortality rates and mean survival time according to phase angle quartiles.

## Abbreviations

The following abbreviations are used in this manuscript:

Alb	Albumin
AUC	Area Under the Curve
BIA	Bioelectrical Impedance Analysis
BUN	Blood Urea Nitrogen
Cl	Chloride
CKD	Chronic Kidney Disease
Cr	Creatinine
CRP	C-Reactive Protein
CVD	Cardiovascular Disease
DM	Diabetes Mellitus
ECW/ICW	Extracellular water to intracellular water ratio
GNRI	Geriatric Nutritional Risk Index
Hb	Hemoglobin
HD	Hemodialysis
HDL-C	High-density Lipoprotein Cholesterol
iPTH	Intact Parathyroid Hormone
Kt/V	Urea Clearance Normalized by Distribution Volume
Na	Sodium
NT-proBNP	*N*-terminal Pro-Brain Natriuretic Peptide
PA	Phase Angle
PEW	Protein-Energy Wasting
QOL	Quality of Life
ROC	Receiver Operating Characteristic
SF-36	36-Item Short Form Health Survey
UA	Uric Acid
β2MG	Beta-2 Microglobulin
