# Supplementary material for: Clinical Significance of Phase Angle for Assessing Quality of Life and Prognosis in Hemodialysis Patients
Source: Nutrients. 2025 Nov 21;17(23):3631. doi: 10.3390/nu17233631 (PMC12693557; doi:10.3390/nu17233631)
Supplement: Supplementary file 1 [file nutrients-17-03631-s001.zip › nutrients-3956361-supplementary.pdf]

Supplementary

Table S1. Associations of Phase Angle with QOL Scores in Multiple Linear Regression Models

QOL Items	Model	β (SE)	*P* score
Physical Functioning	Model 1	9.34 (1.45)	<0.001
	Model 2	8.35 (1.94)	<0.001
	Model 3	8.31 (2.10)	<0.001
Role Physical	Model 1	12.88 (2.51)	<0.001
	Model 2	9.59 (3.35)	0.005
	Model 3	8.46 (3.63)	0.020
Bodily Pain	Model 1	5.65 (1.64)	<0.001
	Model 2	4.74 (2.20)	0.032
	Model 3	3.65 (2.43)	0.133
General Health	Model 1	2.99 (1.15)	0.010
	Model 2	5.92 (1.50)	<0.001
	Model 3	4.52 (1.63)	0.006
Vitality	Model 1	4.05 (1.45)	0.006
	Model 2	5.67 (1.91)	0.003
	Model 3	3.48 (2.09)	0.003
Social Functioning	Model 1	4.25 (1.78)	0.020
	Model 2	3.26 (2.40)	0.174
	Model 3	2.18 (2.67)	0.414
Role Emotional	Model 1	11.94 (2.64)	<0.001
	Model 2	7.99 (3.52)	0.024
	Model 3	6.54 (3.88)	0.093
Mental Health	Model 1	2.75 (1.45)	0.060
	Model 2	2.47 (1.95)	0.208
	Model 3	0.95 (2.15)	0.659
Symptoms	Model 1	1.39 (1.35)	0.305
	Model 2	0.18 (2.25)	0.935
	Model 3	-0.68 (2.23)	0.760
Effects of Kidney Disease	Model 1	2.87 (1.42)	0.043
	Model 2	0.52 (2.34)	0.824
	Model 3	0.17 (2.35)	0.941
Burden of Kidney Disease	Model 1	1.39 (1.40)	0.324

	Model 2	-0.45 (2.23)	0.838
	Model 3	-0.37 (2.28)	0.870
Work Status	Model 1	13.54 (2.25)	<0.001
	Model 2	4.13 (3.49)	0.238
	Model 3	8.37 (3.67)	0.023
Cognitive Function	Model 1	0.68 (1.45)	0.639
	Model 2	-1.21 (2.42)	0.617
	Model 3	-2.22 (2.40)	0.356
Quality of Social Interaction	Model 1	0.57 (1.53)	0.709
	Model 2	0.16 (2.48)	0.948
	Model 3	0.56 (2.52)	0.824
Sleep	Model 1	-0.005 (1.34)	0.997
	Model 2	-0.86 (2.21)	0.695
	Model 3	-0.85 (2.22)	0.701
Social Support	Model 1	-0.77 (1.56)	0.623
	Model 2	0.38 (2.58)	0.883
	Model 3	-0.93 (2.63)	0.726
Dialysis Staff Encouragement	Model 1	-0.21 (1.57)	0.892
	Model 2	-1.30 (2.54)	0.607
	Model 3	-2.68 (2.58)	0.299
Patient Satisfaction	Model 1	0.54 (1.37)	0.694
	Model 2	-1.45 (2.26)	0.528
	Model 3	-2.32 (2.28)	0.309

Note: β coefficients (SE) were estimated to use linear regression models with phase angle as the predictor. Model 1 is univariate; Model 2 is adjusted for age, sex, and NT-proBNP; Model 3 is further adjusted for dialysis duration, diabetes mellitus, cardiovascular disease, smoking status, serum creatinine, hemoglobin, GNRI, Kt/V, and CRP.

Table S2. Mortality Rate and Mean Survival Time According to Phase Angle Quartiles

Group (Phase angle quartile)	Number of events (Deaths)	Censored	Mean survival time (days)	Mortality rate per 100 person-years
Q1	21	49	1198.4	9.14
Q2	7	68	946.7	2.81

Q3	2	76	1323.4	0.71
Q4	4	68	1344.7	1.51

Note: Data is stratified by PA quartiles. Mortality rates are expressed per 100 person-years. Patients in Q1 had the highest mortality rate and the shortest mean survival time, whereas Q3 had the lowest mortality rate.

Figure S1. Receiver Operating Characteristic (ROC) Curve of Phase Angle for Predicting All-Cause Mortality in Female Patients

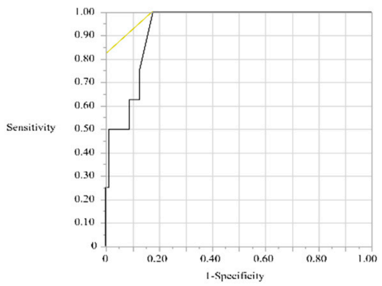

Note: This ROC curve illustrates the discriminatory ability of whole-body PA to predict all-cause mortality in female patients. The AUC was 0.932, indicating excellent predictive performance. The optimal cutoff value for PA, determined by the point at which the sum of sensitivity and specificity was maximized, was 4.0. At this cutoff, sensitivity and specificity were 100% and 82.3%, respectively.

Figure S2. Receiver Operating Characteristic (ROC) Curve of Phase Angle for Predicting All-Cause Mortality in Male Patients

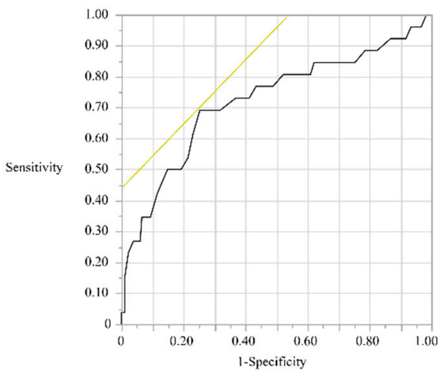

Note: This ROC curve shows the predictive accuracy of PA for all-cause mortality in male patients.

The AUC was 0.722, reflecting fair discrimination. The optimal PA cutoff value was identified as 4.8, with corresponding sensitivity of 69.2% and specificity of 74.2%.

Table S3. Adjusted Hazard Ratios for All-Cause Mortality According to Phase Angle Quartiles (Q1 as Reference) Based on a Parametric Survival Model.

**Model**	**Comparison**	**Hazard Ratio**	**95% CI**	***p*-value**
Model 1	Q2 vs Q1	4.21	2.14–8.29	<0.001
	Q3 vs Q1	14.59	5.89–36.16	<0.001
	Q4 vs Q1	6.73	3.51–12.90	<0.001
Model 2	Q2 vs Q1	3.1	1.50–6.42	0.002
	Q3 vs Q1	9.37	3.76–23.36	<0.001
	Q4 vs Q1	2.97	1.52–5.80	0.001
Model 3	Q2 vs Q1	3.59	1.54–8.35	0.002
	Q3 vs Q1	11.7	4.10–33.37	<0.001
	Q4 vs Q1	3.96	1.74–8.99	0.001

Note: Hazard ratios (HRs) and 95% confidence intervals (CIs) were estimated using a Weibull distribution model. HRs represent the mortality risk for each phase angle (PA) quartile compared to Q1 (reference). Model 1 is univariate; Model 2 is adjusted for age, sex, and NT-proBNP; Model 3 is further adjusted for dialysis duration, diabetes mellitus, cardiovascular disease, smoking status, baseline serum creatinine, hemoglobin, GNRI, Kt/V, and CRP.
